# Automated segmentation of head CT scans for computer-assisted craniomaxillofacial surgery applying a hierarchical patch-based stack of convolutional neural networks

**DOI:** 10.1007/s11548-022-02673-5

**Published:** 2022-06-03

**Authors:** David Steybe, Philipp Poxleitner, Marc Christian Metzger, Leonard Simon Brandenburg, Rainer Schmelzeisen, Fabian Bamberg, Phuong Hien Tran, Elias Kellner, Marco Reisert, Maximilian Frederik Russe

**Affiliations:** 1 Department of Oral and Maxillofacial Surgery, Medical Center – University of Freiburg, Faculty of Medicine, University of Freiburg, Hugstetter Str. 55, 79106 Freiburg, Germany; 2 Berta-Ottenstein-Programme for Clinician Scientists, Faculty of Medicine, University of Freiburg, Freiburg, Germany; 3 Department of Diagnostic and Interventional Radiology, Medical Center – University of Freiburg, Faculty of Medicine, University of Freiburg, Freiburg, Germany; 4 Department of Medical Physics, Medical Center – University of Freiburg, Faculty of Medicine, University of Freiburg, Freiburg, Germany

**Keywords:** Computer-assisted surgery, Craniomaxillofacial surgery, Deep learning, Convolutional neural networks, Medical image segmentation

## Abstract

**Purpose:**

Computer-assisted techniques play an important role in craniomaxillofacial surgery. As segmentation of three-dimensional medical imaging represents a cornerstone for these procedures, the present study was aiming at investigating a deep learning approach for automated segmentation of head CT scans.

**Methods:**

The deep learning approach of this study was based on the patchwork toolbox, using a multiscale stack of 3D convolutional neural networks. The images were split into nested patches using a fixed 3D matrix size with decreasing physical size in a pyramid format of four scale depths. Manual segmentation of 18 craniomaxillofacial structures was performed in 20 CT scans, of which 15 were used for the training of the deep learning network and five were used for validation of the results of automated segmentation. Segmentation accuracy was evaluated by Dice similarity coefficient (DSC), surface DSC, 95% Hausdorff distance (95HD) and average symmetric surface distance (ASSD).

**Results:**

Mean for DSC was 0.81 ± 0.13 (range: 0.61 [mental foramen] – 0.98 [mandible]). Mean Surface DSC was 0.94 ± 0.06 (range: 0.87 [mental foramen] – 0.99 [mandible]), with values > 0.9 for all structures but the mental foramen. Mean 95HD was 1.93 ± 2.05 mm (range: 1.00 [mandible] – 4.12 mm [maxillary sinus]) and for ASSD, a mean of 0.42 ± 0.44 mm (range: 0.09 [mandible] – 1.19 mm [mental foramen]) was found, with values < 1 mm for all structures but the mental foramen.

**Conclusion:**

In this study, high accuracy of automated segmentation of a variety of craniomaxillofacial structures could be demonstrated, suggesting this approach to be suitable for the incorporation into a computer-assisted craniomaxillofacial surgery workflow. The small amount of training data required and the flexibility of an open source-based network architecture enable a broad variety of clinical and research applications.

**Supplementary Information:**

The online version contains supplementary material available at 10.1007/s11548-022-02673-5.

## Introduction

Computer-assisted procedures represent an essential component of contemporary craniomaxillofacial surgery: Virtual three-dimensional datasets enable detailed preoperative planning of resection and/or reconstruction in head and neck cancer and trauma and provide the basis for the fabrication of patient-specific cutting guides and patient specific implants [1, 2]. Moreover, computer-assisted planning can aid in preoperatively determining the optimal surgical approach, especially in highly complex regions such as the skull base [3]. Intraoperatively, a variety of procedures, including pre-planned resection and reconstruction, can be realized and controlled by applying CAD/CAM fabricated guides and implants, navigated surgery, or a combination of both approaches [4, 5]. Subsequently, comparing volumetric and morphological features in the patient's preoperative planning and intra-/postoperative imaging facilitates immediate evaluation of the surgical outcome [6, 7]. All of these procedures are based on three-dimensional volume and surface reconstructions, which makes segmentation of the respective structures of interest in three-dimensional medical imaging a foundation for computer-assisted craniomaxillofacial surgery.

For many years, medical image segmentation has been a labor intensive manual process, and the advent of (semi)automated atlas-or model-based segmentation approaches was a first major evolution in this context [8]. In the recent past, Artificial intelligence (AI) applications have started to revolutionize a variety of medical fields, including medical image segmentation [9, 10]. Most commonly, the networks applied for automated image segmentation are based on the U-Net, which, in its original version, is a general-purpose segmentation network for 2D images. It was inspired by the fully convolutional networks [11], but uses a downsampling and upsampling structure as well as skip connection to reduce the number of parameters and computation time required in comparison to previous convolutional neural network (CNN) architectures [12]. Based on this work, many adaptations have been developed specifically for 3D medical image segmentation, such as the 3D U-Net or the V-Net, which are addressing segmentation problems in cross-sectional medical image data [13, 14]**.**

While AI-based segmentation of organs at risk in radiation therapy planning for the head and neck region has been reported by a number of investigators [15], to date, only little attention has been paid to AI-based segmentation in the field of computer-assisted craniomaxillofacial surgery [16]. In this context, it was the aim of this study to investigate the application of a hierarchical patch-based stack of CNNs based on the U-Net architecture, for automated segmentation of bone structures (viscerocranium, skull base, mandible), foramina/canals, paranasal sinuses and soft tissue (ocular globe, optic nerve, extraocular muscles).

## Materials and methods

### Structure of the segmentation network

For this project, the patchwork toolbox (https://bitbucket.org/reisert/patchwork/), which employs a multiscale stack of 3D convolutional neural networks, was chosen as the basis for deep learning based segmentation. The settings applied mainly consisted of the default parameters of this network, which had been selected when the toolbox was created on several datasets such as the Medical Segmentation Decathlon, which focuses on generalizable 3D semantic segmentation [17].

The framework uses nested patches of fixed matrix size, but decreasing physical size. In each scale, a U-Net-type architecture was used, where the matrix size of the U-Net was always of size 32^3^ voxels for all scales and a scale pyramid of depth four was applied. These settings were based on the available hardware capacity, which was the limiting factor for sample size and pyramid size. The size of the pyramid was selected such that it allowed for as much 3D field-of-view as possible (80% of the image) in the coarsest layer and a very high spatial resolution in the smallest layer with an isotropic resolution of 1 mm. The matrix size was selected such that it would map representative portions of the anatomy.

**Fig. 1:**
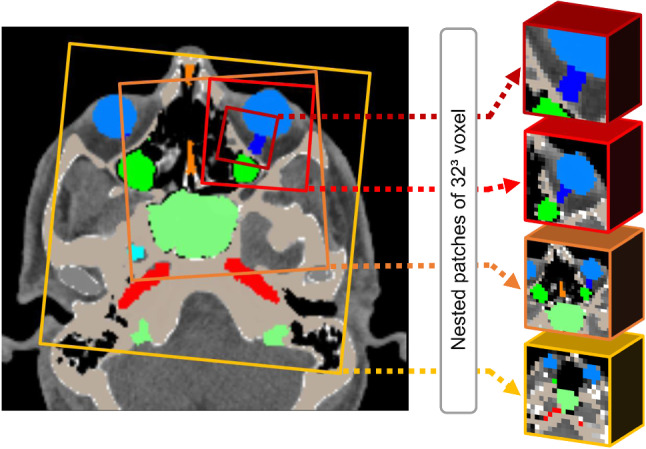
Simplified representation of the patching strategy: Nested patches are created in four levels, which each have a fixed matrix size of 32*32*32 voxels, while image detail increases over the levels. The patches are drawn randomly within each other, with augmentation of each patch for network training

The scale of the intermediate levels were exponentially interpolated, such that the view of the labels would occur in reasonable resolution steps. A simplified visualization of the patching strategy is presented in Fig. 1, were the reformation of the labeled images based on patching sizes is visualized as well.

The input to the network is the CT image in HU units. Instead of normalizing/clipping the image, an initial channel splitting layer was employed. This channel splitting layer separated the input range into 11 feature channels sensitive to a certain HU-range. This method was inspired by the windowing approach used by a radiologist during image reading, separating the total HU-range into solvable image parts e.g., CT window for soft tissue or bone. The architecture of the basis U-Net applied in this project is close to the default UNet configuration as presented in the literature [12, 13], with feature dimensions (8,16,16,32,64) and max-pooling in the encoding layers and transposed convolutions in the decoding layers. Each U-Net has n+8 output channels, where the first n are corresponding to the labels and are used for intermediate loss computations. The logits of the total n+8 outputs are just forwarded as input to the next scale. The network is trained with the Adam optimizer [18] with rate 0.001. No systematic tuning was done, as this was assumed to result in a significant extension of training time. Only an adaptation of loss function was undertaken, based on expected problems for small labels: Labels large in volume were trained using ordinary softmax activation and categorical cross-entropy [19] as loss function, while for small labels, a binary-crossentropy variant of the top-K loss was employed [20, 21]. For each label, the voxels with the K largest loss values were selected for loss computation.

### Dataset and settings

A total of 18 craniomaxillofacial structures were segmented as part of this study; these included bone structures as well as soft tissue structures and small regions of interest like foramina and canals (Table 1). The CT scans included in this study were retrieved from the Picture Archiving and Communication System (PACS) of the University Medical Center Freiburg. The scans included were limited to those with a slice thickness < 1 mm and reconstructed with the Br36 or Br40 algorithm. For computing efficiency and better comparability of results, image resolution was resized to a voxel size of 1 mm^3^. Only scans of patients without affections of the segmented structures were included. For ground truth, the dataset was segmented manually by a physician trained in head and neck image segmentation (DS). Manual segmentation was performed on the NORA imaging platform (https://www.nora-imaging.com), applying shape- and threshold-based tools. All manual segmentations were carefully reviewed by a board certified radiologist (MFR).

**Table 1:** Results for quantification of segmentation accuracy (Patchwork network) by DSC, Surface DSC, 95HD and ASSD for all structures and groups evaluated in the present study

		Dice similarity coefficient (mean ± SD)	Surface dice similarity coefficient (mean ± SD)	95% Hausdorff distance (mean ± SD in mm)	Average symmetric surface distance (mean ± SD in mm)
Bones	Viscerocranium/skull base	0.94 ± 0.02	0.98 ± 0.02	1.08 ± 0.17	0.12 ± 0.06
	Nasal septum	0.86 ± 0.04	0.93 ± 0.06	3.20 ± 2.92	0.62 ± 0.46
	Mandible	0.98 ± 0.01	0.99 ± 0.01	1.00 ± 0.00	0.09 ± 0.01
	*Bones (all)*	*0.93* ± *0.06*	*0.97* ± *0.04*	*1.76* ± *2.04*	*0.28* ± *0.37*
Sinuses	Frontal sinus	0.93 ± 0.01	0.96 ± 0.03	1.61 ± 0.77	0.22 ± 0.11
	Sphenoid sinus	0.93 ± 0.02	0.94 ± 0.02	2.29 ± 1.08	0.38 ± 0.13
	Maxillary sinus	0.94 ± 0.06	0.95 ± 0.08	4.12 ± 6.25	0.16 ± 0.05
	*Sinuses (all)*	*0.93* ± *0.04*	*0.95* ± *0.05*	*2.67* ± *3.97*	*0.25* ± *0.14*
Canals	Nasolacrimal duct	0.81 ± 0.03	0.98 ± 0.01	1.00 ± 0.00	0.28 ± 0.07
	Carotid canal	0.80 ± 0.08	0.91 ± 0.09	1.83 ± 1.02	0.49 ± 0.32
	Jugular foramen	0.83 ± 0.03	0.93 ± 0.04	1.48 ± 0.13	0.46 ± 0.08
	*Canals (all)*	*0.81* ± *0.06*	*0.94* ± *0.07*	*1.44* ± *0.71*	*0.41* ± *0.22*
Foramina	Foramen ovale	0.80 ± 0.02	0.98 ± 0.02	1.17 ± 0.20	0.29 ± 0.05
	Foramen rotundum	0.65 ± 0.11	0.95 ± 0.05	1.28 ± 0.39	0.40 ± 0.18
	Foramen spinosum	0.65 ± 0.07	0.95 ± 0.04	1.43 ± 0.40	0.41 ± 0.12
	Infraorbital foramen	0.68 ± 0.09	0.91 ± 0.07	3.56 ± 2.47	0.55 ± 0.44
	Mandibular foramen	0.72 ± 0.06	0.94 ± 0.05	1.54 ± 0.48	0.44 ± 0.18
	Mental foramen	0.61 ± 0.11	0.87 ± 0.08	2.44 ± 1.28	1.19 ± 1.28
	*Foramina (all)*	*0.68* ± *0.10*	*0.93* ± *0.07*	*1.90* ± *1.48*	*0.55* ± *0.65*
Soft tissue	Ocular globe	0.93 ± 0.01	0.95 ± 0.03	1.25 ± 0.20	0.38 ± 0.08
	Extraocular muscles	0.76 ± 0.05	0.93 ± 0.05	2.25 ± 1.25	0.55 ± 0.27
	Optic nerve	0.75 ± 0.05	0.93 ± 0.03	2.20 ± 0.75	0.50 ± 0.17
	*Soft tissue (all)*	*0.81* ± *0.10*	*0.93* ± *0.04*	*1.90* ± *1.00*	*0.48* ± *0.21*
	***All structures***	***0.81*** ± ***0.13***	***0.94*** ± ***0.06***	***1.93*** ± ***2.05***	***0.42*** ± ***0.44***

The created labels were divided into 5 groups to reflect the different types of annotated structures (Table 1), and a multiclass network was trained for each group. Each class of labels was unique and when present, contained the structure of both sides. Within each training task, patches were created utilizing the defined patch strategy. Prior to feeding the patches to the network for training, a random augmentation was performed, allowing rotation up to 10°, a horizontal flip and image scaling up to 20%.

Each Patchwork CNN was trained for 2 million patches. Training time per patch was around 19–25 ms with additional time for loading the patch and saving the learned network; the training per network took around 20–24 h. Training was performed on a GPU accelerated server system using a Nvidia RTX (A)6000 graphic unit. During training, patches were randomly sampled such that approximately 80% of the finest patches contained at least one label.

For creating the results in the separate validation dataset, a random patching scheme was used, where for each promising patch, three patches of the following level were selected. The prediction of a full volume was performed on a 16-core CPU machine (without a GPU) which, correspondingly to the used image volume, took several minutes.

For comparison of the segmentation approach reported in this paper, the nnUNet [22], as one of the state-of-the-art self-regulating networks for medical image segmentation, was applied to the dataset used in this study. The 3d full resolution U-Net variant, which was considered the approach of the nnUNet framework with highest comparability to the approach reported in the present study, was chosen. Training duration was around 24 h using a NVIDA RTX A6000; however, when comparing this to the Patchwork segmentation approach, it has to be considered that the nnUNet is equipped with additional acceleration methods like mixed precision and optimized parallelization methods, which are not yet included in the Patchwork network.

### Evaluation metrics

For quantitative evaluation of the performance of the segmentation network, segmentations generated by the automated approach were compared to manual segmentations applying a separate validation dataset consisting of five CT scans. The following evaluation metrics were applied:

Dice similarity coefficient (DSC): The DSC is a spatial overlap index ranging from 0, indicating no spatial overlap between two sets of binary segmentation results, to 1, indicating complete overlap [23].

Surface Dice similarity coefficient (Surface DSC): The surface DSC quantifies the deviation between the surface contours of the selected structures. An acceptable tolerance of 1 mm was chosen, as this corresponds to a displacement of 1 voxel at the used image data resolution [24].

Hausdorff distance (HD): The HD measures the maximum surface distance in millimeters between segmentations. In this study, the “robust” Hausdorff distance was applied because it uses the 95th percentile (95HD) of the distances to be more robust against small outliers [3]**.**

Average symmetric surface distance (ASSD): The ASSD calculates the distances between two surfaces. It works like the Hausdorff distance, but uses the minimum distance instead of the maximum distance and therefore does not penalize outliers as much as the Hausdorff distance [25].

## Results

**Fig. 2:**
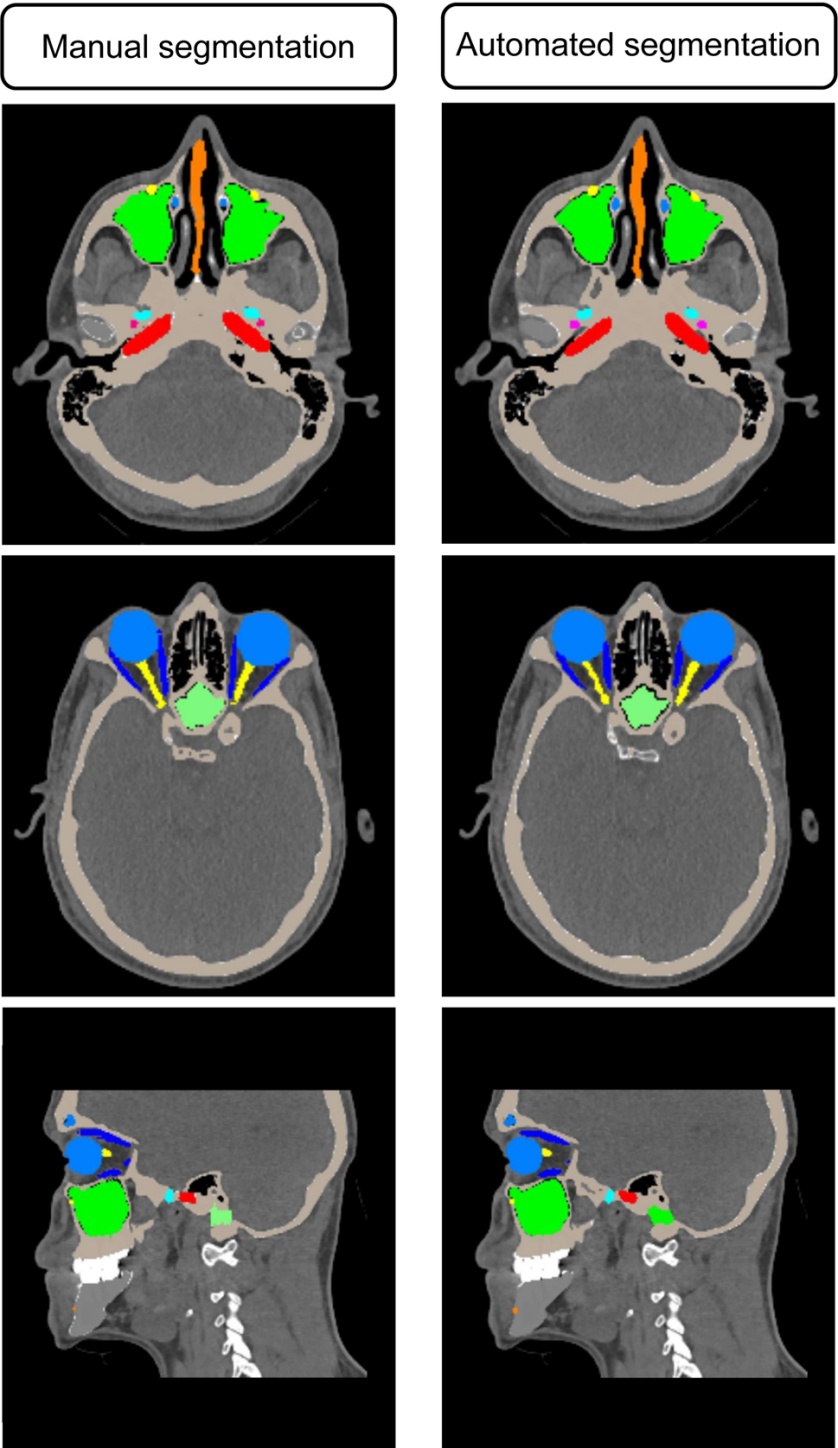
2D views of results of manual and automated segmentation in corresponding slices of the validation dataset

**Fig. 3:**
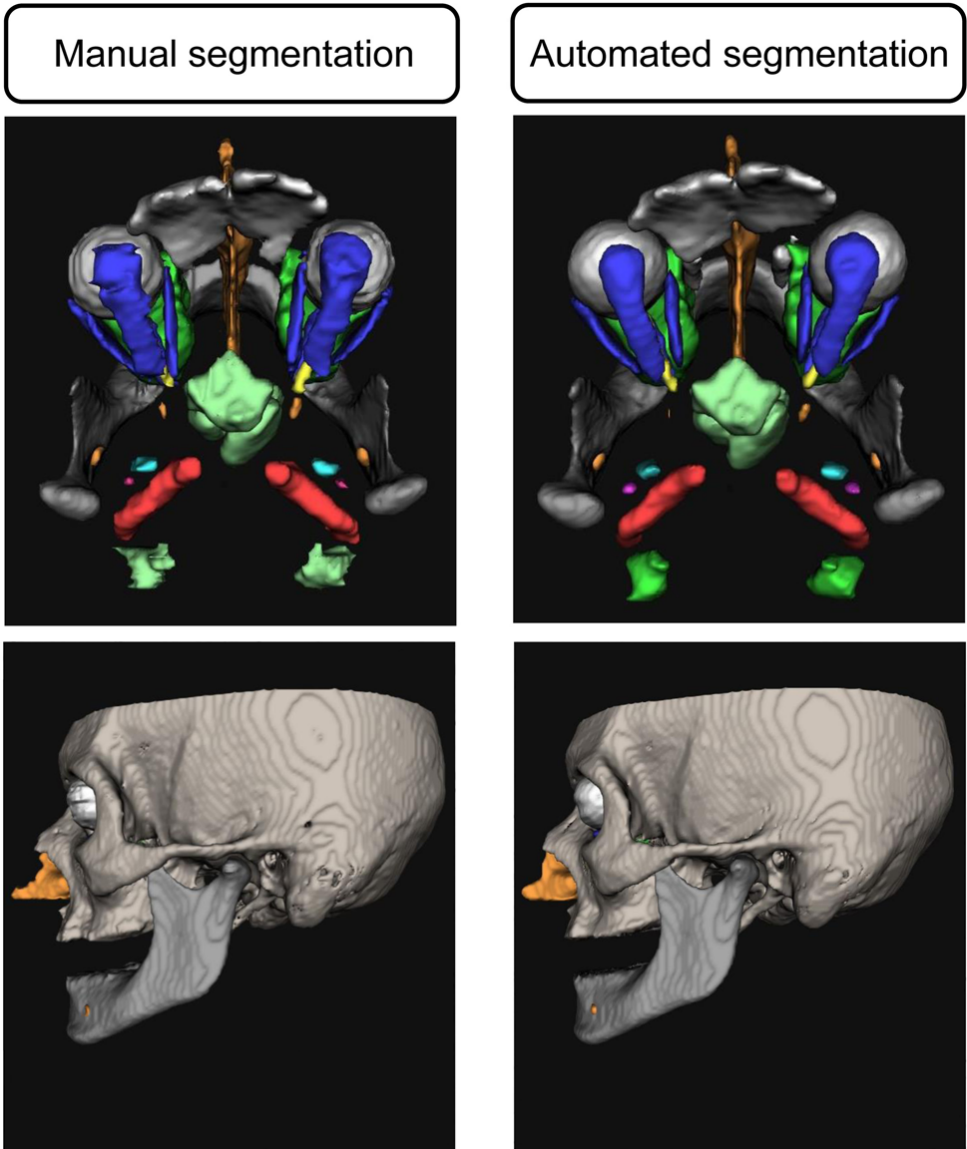
3D reconstructions of results of manual and automated segmentation from a CT scan of the validation dataset

**Fig. 4:**
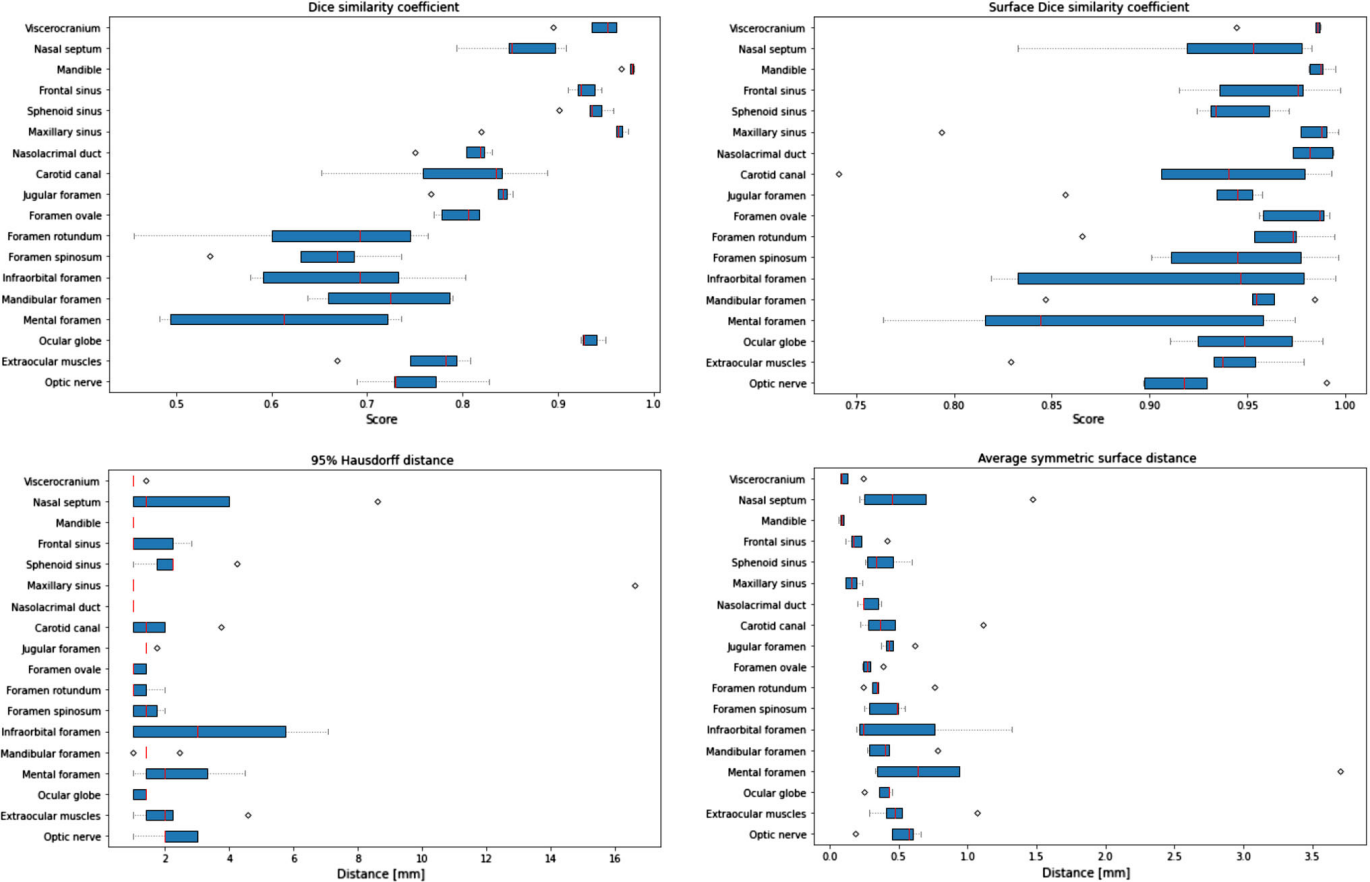
Box-plots of segmentation accuracy (DSC, Surface DSC, 95HD and ASSD) for all structures and groups evaluated in the present study

For evaluation of the accuracy of the automated approach, segmentation results were compared to manual (ground truth) segmentations in a validation dataset consisting of five CT scans. Figure 2 shows 2D views, and Fig. 3 shows 3D reconstructions of segmentations performed in the validation dataset by the automated approach and the corresponding manual segmentations. Box-plots for all structures and evaluation metrics applied in this study are presented in Fig. 4. An overview of mean values and standard deviations can be found in Table 1.

For the Patchwork network, the mean DSC was found to range from 0.61 (mental foramen) to 0.98 (mandible), with a mean value of 0.81 ± 0.13 considering all structures. While values exceeding 0.9 were found for the mandible, viscerocranium/skull base, sinuses and ocular globe, lower values were found for the other structures included in this study. For the Surface DSC, mean values ranging from 0.87 (mental foramen) to 0.99 (mandible) (mean 0.94 ± 0.06) were obtained, with values exceeding 0.9 for all structures but the mental foramen (0.87). The mean 95HD had a range of 1.00 (mandible) to 4.12 mm (maxillary sinus) (mean 1.93 ± 2.05 mm), with values less than 2 mm for all structures but the optic nerve, extraocular muscles, sphenoid sinus, mental foramen, nasal septum, infraorbital foramen and maxillary sinus. For the ASSD, mean values ranging from 0.09 (mandible) to 1.19 mm (mental foramen) (mean 0.42 ± 0.44) were found, with values of less than 1 mm for all structures but the mental foramen.

Applying the trained 3d full resolution U-Net from nnUNet to the validation dataset, all labels beside the infraorbital and mental foramen were predicted. Considering all anatomical structures included in this study, a mean DSC of 0.74 ± 0.28 (Patchwork: 0.81 ± 0.13) was found, ranging from 0.65 (foramen rotundum) to 0.98 (mandible). For the Surface DSC a mean of 0.84 ± 0.30 (Patchwork: 0.94 ± 0.06) was obtained, ranging from 0.9 (frontal sinus) to 0.99 (mandible). Regarding the labels that were found by the nnUNet, results were comparable to the Patchwork network (Online Resource 1).

## Discussion

Segmentation of head CT data represents a cornerstone for computer-assisted craniomaxillofacial surgery; however, to date only little attention has been paid to AI-based approaches in this field [16]. In this study, a hierarchical patch-based stack of CNNs based on the U-Net architecture was applied for the purpose of automated head CT segmentation. A major characteristic of this architecture is that the image data are processed patch-wise in connected levels of image resolution and thus the dependence on image size and image matrix plays a subordinate role (https://bitbucket.org/reisert/patchwork/). This approach accounts for the fact that in medical imaging, the coverage may vary based on scan length and field of view and addresses a major drawback of many deep learning architectures, which require a consistent image matrix of the imported data to achieve optimal results. In addition, the network has the ability to learn anatomical relationships, such as that the maxillary sinus lies below the frontal sinus, while still accounting for the geometric variability of the edge structure. Moreover, the hierarchical approach allows to avoid anatomically nonsensical outliers despite the small sample size. As expected, the applied technique of balancing and patch based sampling of the data helped to represent small labels in the training task, with none of the labels missed in the performed prediction. Comparing the Patchwork network to the nnUNet framework, which missed two of the labels included in this study, under the exclusion of these two labels near identical results were obtained by the Patchwork network (DSC: 0.83 ± 0.12; Surface DSC: 0.95 ± 0.05) and the nnUNet (DSC: 0.83 ± 0.11; Surface DSC: 0.95 ± 0.05). As both networks consist of UNet structures with an isotropic resolution of up to 1 mm^3^ and an adaptive cropping method, this result with only minor differences for the larger labels is what could be expected.

Reviewing the literature on other CNN based segmentation approaches, which are based on much larger datasets in the majority of cases (Table 2), it could be demonstrated that the approach applied in this present study performed competitively as well.

**Table 2:** Results reported in recent literature for structures segmented in the present study

	Mandible				Maxillary sinus	Ocular globe		Optic nerve		
	DSC	Surface DSC	95HD (mm)	ASSD (mm)	DSC	DSC	95HD (mm)	DSC	Surface DSC	95HD (mm)
[26]	97.59 ± 0.43%		0.491 ± 0.021	0.065 ± 0.020						
[27]	97.48%		2.656	0.217						
[24]		95.5%							97.5%/ 98.8%	
[28]					94.40 ± 2.07%					
[29]						94%/ 93.5%		80.3%/ 82.2%		
[30]						0.95	1.5			
[31]								0.71 ± 0.08		2.23 ± 0.90
*Present study*	*0.98* ± *0.01*	*0.99* ± *0.01*	*1.00* ± *0.00*	*0.09* ± *0.01*	*0.94* ± *0.06*	*0.93* ± *0.01*	*1.25* ± *0.20*	*0.75* ± *0.05*	*0.93* ± *0.03*	*2.20* ± *0.75*

The surface DSC, which measures the offset of the delineated structures, revealed high accuracy (> 0.9) for all structures but the mental foramen. The volumetric DSC on the other hand revealed high segmentation accuracy for large structures like the viscerocranium/skull base, mandible, sinuses and ocular globe, but yielded less accurate results for small structures, e.g., foramina. This finding can be explained by the fact that the volumetric DSC compares the volumetric overlap of segmentations and therefore has a bias toward large structures, arising from internal volumes [24]. Besides the DSC and surface DSC, the 95HD and the ASSD were applied for quantitative evaluation of the accuracy of automated segmentation. Although the 95HD is reported frequently in the literature and thus was included as a metric in this study to provide comparability, the more modern ASSD, just like the Surface DSC, better reflects the amount of supervision and manual correction required to align the automated segmentations to ground truth. Measuring the offset of the segmentations in millimeters, the ASSD gives a good estimate of the range of the boundaries the segmented structures can be expected in and thus can be considered to provide an appropriate assessment of the clinical utility of the segmentations generated by the automated approach. It can be assumed that the high accuracy of automated segmentation demonstrated for the network applied in this study makes this approach suitable for the application in computer-assisted craniomaxillofacial surgery. Even in case of less accurate segmentation results, which were found for some of the smaller structures in this study, e.g., foramina, the fact that all of these structures were identified in all cases provides the option of defining “danger zones” of a few millimeters, which can support intraoperative orientation. However, validating these assumptions was not within the scope of this work, which had a primarily technical focus.

It has to be considered that in this study segmentation and validation were performed in CT-scans without pathologies. Thus, it can be expected that altered anatomical structures, e.g., due to trauma or tumors, would affect segmentation accuracy of the current model. However, once CT-scans of trauma and/or tumor cases are included in the training data in a representative manner, it can be expected that fast adaptation of the network to these pathologies will be possible, although it has to be considered that some pathological changes may be unique and therefore challenging for AI.

Moreover, it might be considered a shortcoming that manual segmentation of the dataset was performed by a single investigator. However, all manual segmentations were reviewed carefully by a second observer (board certified radiologist).

Improved accuracy and predictability as well as reduced duration of surgery have been demonstrated with the application of computer-assisted procedures in craniomaxillofacial surgery [32–34]. In this context, automated segmentation of CT data can facilitate a computer-assisted workflow of preoperative virtual surgical planning, CAD/CAM-assisted and/or navigated surgery and postoperative outcome verification [16]. While precise segmentation of bone (mandible and viscerocranium/skull base) represents the outset for virtual planning in most cases, segmenting further soft and hard tissue structures provides valuable additional information for computer-assisted planning and surgery. Mapping of critical structures like nerves and vessels for instance can provide valuable intraoperative information to the surgeon [35]**.** In reconstruction of complex defects of the orbit, 3D-data including nerves and oculomotor muscles can provide additional information for construction of patient specific implants (PSI) and for accurate placement of such implants [36]. As another example, in septoplasty automated segmentation can facilitate virtual surgery planning, which has the potential to improve surgical outcomes [37]**.** Moreover, automated image segmentation could make a major contribution to the field of statistical shape models (SMM) in craniomaxillofacial surgery. The application of SMMs has been reported frequently and demonstrated versatile usability for different research and clinical applications [38, 39]; however, as of today, a shortcoming of this approach is the amount of data incorporated into a specific SSM due to limited availability of segmented image data. Last but not least, it can be expected that computer-assisted craniomaxillofacial surgery will be further promoted by the fast progress made in augmented/virtual reality applications in this field [40] and taking full advantage of these developments once more demands detailed and precise medical image segmentation.

## Conclusion

The high accuracy of automated segmentation of a variety of craniomaxillofacial structures, together with the fact that none of the small labels had been missed by the deep learning segmentation network reported in this article, suggest this approach to be suitable for the incorporation into a computer-assisted craniomaxillofacial surgery workflow. The small amount of training data required to obtain high quality segmentations and the flexibility of an open source based network architecture enable a broad variety of future research and clinical applications.

## Supplementary Information

Below is the link to the electronic supplementary material.

Supplementary file1 (PDF 30 KB)

## Data Availability

The Patchwork codebase applied in this work is available as a git-repo on Bitbucket (https://bitbucket.org/reisert/patchwork/). The trained model can be made available upon request.
