# Supplementary material for: Automated segmentation of head CT scans for computer-assisted craniomaxillofacial surgery applying a hierarchical patch-based stack of convolutional neural networks
Source: Int J Comput Assist Radiol Surg. 2022 Jun 3;17(11):2093–101. doi: 10.1007/s11548-022-02673-5 (PMC9515026; doi:10.1007/s11548-022-02673-5)
Supplement: Supplementary file 1 — Supplementary file1 (PDF 30 KB) [file 11548_2022_2673_MOESM1_ESM.pdf]

Online Resource 1: Results for quantification of segmentation accuracy (nnUNet) by DSC, Surface DSC, 95HD and ASSD for all structures and groups evaluated in the present study

		**Dice similarity coefficient (mean ± SD)**	**Surface Dice similarity coefficient (mean ± SD)**	**95% Hausdorff distance (mean ± SD in mm)**	**Average symmetric surface distance (mean ± SD in mm)**
Bones	Viscerocranium/skull base	0.95 ± 0.02	0.98 ± 0.02	1.15 ± 0.33	0.16 ± 0.1
	Nasal septum	0.86 ± 0.04	0.93 ± 0.06	3.03 ± 3.87	0.61 ± 0.61
	Mandible	0.98 ± 0.01	0.99 ± 0.003	0.8 ± 0.4	0.07 ± 0.01
	Bones (all)	0.93 ± 0.06	0.97 ± 0.04	1.66 ± 2.33	0.28 ± 0.4
Sinuses	Frontal sinus	0.89 ± 0.08	0.9 ± 0.07	4.64 ± 3.18	0.85 ± 0.70
	Sphenoid sinus	0.93 ± 0.02	0.97 ± 0.03	1.28 ± 0.44	0.19 ± 0.13
	Maxillary sinus	0.95 ± 0.05	0.97 ± 0.07	3.4 ± 5.36	0.15 ± 0.07
	Sinuses (all)	0.93 ± 0.05	0.94 ± 0.07	3.11 ± 3.63	0.4 ± 0.49
Canals	Nasolacrimal duct	0.82 ± 0.05	0.94 ± 0.03	1.48 ± 0.50	0.39 ± 0.16
	Carotid canal	0.82 ± 0.05	0.92 ± 0.06	1.71 ± 0.29	0.48 ± 0.13
	Jugular foramen	0.77 ± 0.05	0.95 ± 0.05	1.6 ± 0.54	0.39 ± 0.13
	Canals (all)	0.8 ± 0.05	0.94 ± 0.05	1.6 ± 0.44	0.42 ± 0.14
Foramina	Foramen ovale	0.82 ± 0.02	0.99 ± 0.01	1 ± 0	0.25 ± 0.04
	Foramen rotundum	0.65 ± 0.09	0.94 ± 0.05	1.6 ± 0.9	0.29 ± 0.06
	Foramen spinosum	0.65 ± 0.03	0.96 ± 0.02	1.25 ± 0.2	0.44 ± 0.14
	Infraorbital foramen	0 ± 0	0 ± 0	*	*
	Mandibular foramen	0.71 ± 0.06	0.94 ± 0.06	1.57 ± 0.82	0.43 ± 0.2
	Mental foramen	0 ± 0	0 ± 0	*	*
	Foramina (all)	0.47 ± 0.34	0.64 ± 0.46	1.35 ± 0.62 **	0.35 ± 0.15 **
Soft tissue	Ocular globe	0.93 ± 0.02	0.94 ± 0.04	1.48 ± 0.50	0.44 ± 0.12
	Extraocular muscles	0.8 ± 0.06	0.96 ± 0.02	1.65 ± 0.77	0.33 ± 0.16
	Optic nerve	0.77 ± 0.06	0.94 ± 0.04	1.8 ± 1.08	0.44 ± 0.2
	Soft tissue (all)	0.83 ± 0.08	0.95 ± 0.04	1.65 ± 0.77	0.4 ± 0.23
	All structures	0.74 ± 0.28	0.84 ± 0.3	1.84 ± 1.98 **	0.37 ± 0.3 **

* Due to lack of predicted label infinity/empty.
** Calculation without missing predictions of infraorbital and mental foramen.
